# When Blood Cell Counts Matter: Hypereosinophilic Syndrome as a Rare Cause of Ischemic Strokes

**DOI:** 10.7759/cureus.60557

**Published:** 2024-05-18

**Authors:** Gunjanpreet Kaur, Wilson Rodriguez, Yoan Ganev, Divya Singh, Adam Awad, Lissette Orozco, Rachel Overberg, Randall C Edgell

**Affiliations:** 1 Neurology, Saint Louis University School of Medicine, St. Louis, USA; 2 Internal Medicine, Trinity Health Ann Arbor Hospital, Ann Arbor, USA

**Keywords:** hypereosinophilia, fip1l1-pdgfra, stroke, thromboembolic strokes, hypereosinophilic syndromes

## Abstract

Hypereosinophilic syndrome (HES) is a rare condition characterized by elevated eosinophil counts (>1.5 x 10^9^ on two consecutive measurements), which are of myeloid clonal in origin or are driven by excess cytokines. One subtype of HES exhibits the Fip1-like 1-platelet-derived growth factor receptor alpha (FIP1L1-PDGFRA) fusion gene, a gain-of-function mutation resulting in a hyperactive tyrosine kinase. HES, especially the FIP1L1-PDGFRA variant, exhibits an excellent response to chemotherapy with imatinib. In this report, we present a 38-year-old patient with no contributory past medical history who experienced sudden-onset fatigue, ataxia, visual changes, and headaches. He was found to have multiple small acute infarcts in his cerebrum and cerebellum. A stroke work-up, including transthoracic echocardiogram (TTE), transesophageal echocardiogram (TEE), and computed tomography angiography (CTA), did not yield insight into the origin of his infarcts. On CBC, he was consistently hypereosinophilic, and a bone marrow biopsy revealed hypercellularity and the FIP1L1-PDGFRA fusion gene, confirming the diagnosis of HES. The patient was treated first with methylprednisolone and then imatinib with excellent response. It appears that, in our patient, strokes were not of a thromboembolic nature but rather due to hypercoagulability. In this report, we advocate for considering HES and emphasize the importance of revisiting basic laboratory studies such as a CBC if the standard stroke workup fails to elucidate the mechanism behind ischemic strokes with an embolic pattern.

## Introduction

Hypereosinophilia can present in a wide variety of contexts, with most cases being classified as primary (coming from a myeloid clone) or secondary, usually traced to parasitic infections, allergies, vasculitis, or drug reactions [1]. When eosinophilia exceeds 1.5 × 10^9^/L for more than six months, after excluding other known causes, and there is evidence of organ dysfunction directly linked to eosinophils, it strongly indicates a diagnosis of hypereosinophilic syndrome (HES) [2]. Some patients present with fatigue and shortness of breath, and many others have cutaneous manifestations and end-organ damage, affecting primarily the lungs, heart, and nervous system [3]. Over half of the patients have a neurological manifestation of the condition, with the most common being embolic strokes, encephalopathy, or peripheral disturbances such as paresthesias [3]. It appears that cardiovascular presentations are more common than neurologic ones, with endomyocardial fibrosis and deep vein thrombosis occurring in more than 60% of patients [4]. While various causes have been documented, in one study, 14% of HES patients exhibited a Fip1-like 1-platelet-derived growth factor receptor alpha (FIP1L1-PDGFRA) fusion gene, due to a deletion in chromosome 4q12, responding very well to low-dose imatinib [5]. In this report, we describe a patient who initially presented with blurred vision, dizziness, and left hemiparesis as a result of bilateral cortical and subcortical infarcts in the setting of HES. A bone marrow biopsy revealed the FIP1L1-PDGFRA gene. He responded excellently to methylprednisolone and imatinib, showing the importance of considering all diagnostic possibilities when the traditional stroke work-up does not yield an answer.

## Case presentation

**Figure 1:**
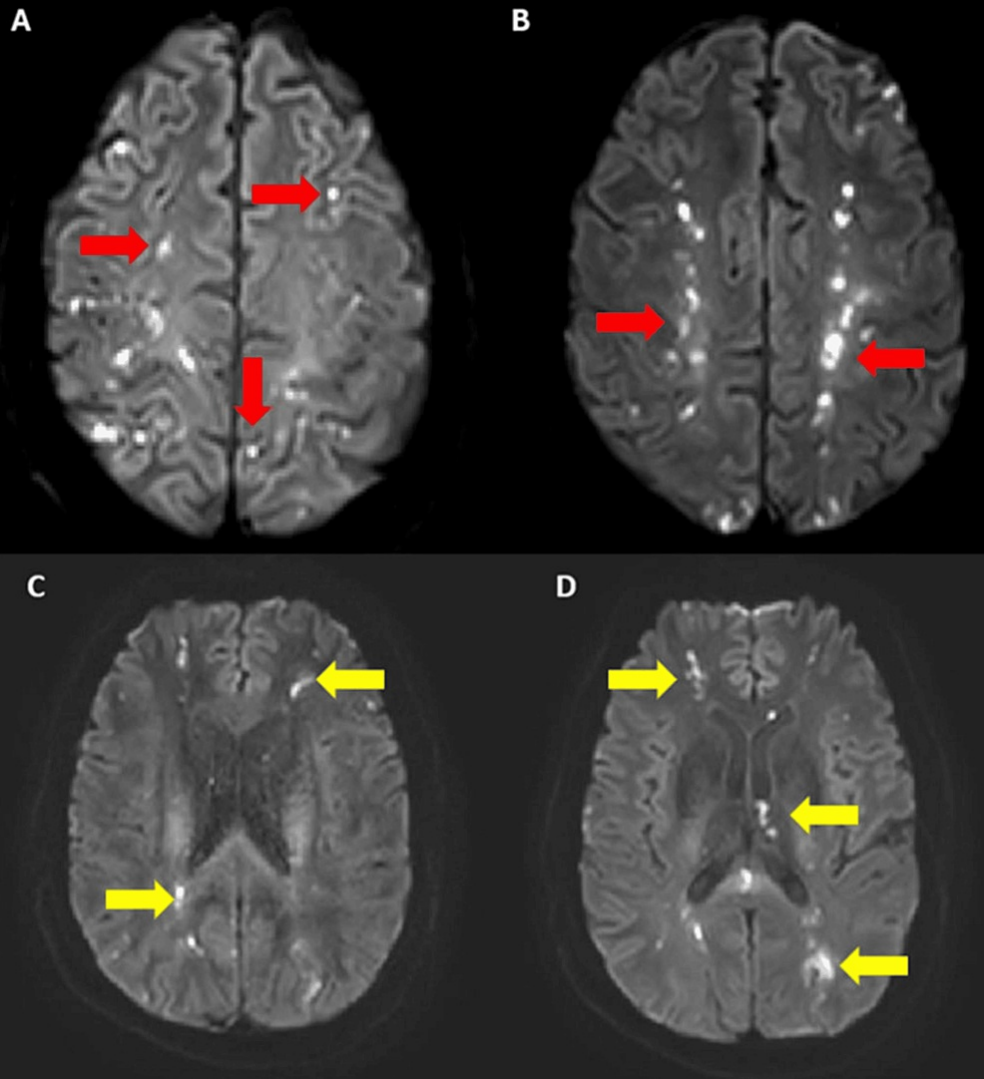
A-B: DWI sequence showing multiple cortical acute infarcts in bilateral hemispheres (red arrows). C-D: sequence showing multiple subcortical acute infarcts in bilateral hemispheres (yellow arrows). Diffusion-weighted imaging

We present the case of a 38-year-old man with a significant medical history of obesity, who experienced sudden-onset visual changes reported as blurred vision in both eyes, ataxia, and dull headaches accompanied by lightheadedness. Upon initial evaluation, his National Institute of Health Stroke Scale (NIHSS) score was 3 due to left limb ataxia, and bilateral drifting of legs. A comprehensive neurological examination revealed left hemiparesis. At an outside institution, imaging revealed multiple cerebral and cerebellar lesions consistent with small acute infarcts (Figure 1). Concurrently, he exhibited marked hypereosinophilia, confirmed by a bone marrow biopsy showing hypercellularity with eosinophil predominance. Treatment with methylprednisolone was initiated, and he was transferred to our institution.

Upon arrival, a repeat MRI revealed numerous multifocal acute infarctions, mainly involving the tentorial region with additional infratentorial region involvement. His eosinophil count on initial complete blood count (CBC) was markedly elevated at 9.05 x 10^9^/L. Further investigation uncovered a deep vein thrombosis (DVT) in the femoral vein, extending to the popliteal, tibioperoneal trunk, and peroneal veins. Additionally, a computed tomography angiography (CTA) of the brain revealed no significant intracranial stenosis, while both transthoracic echocardiogram (TTE) and transesophageal echocardiogram (TEE) did not identify a cardioembolic source of the stroke or significant structural heart defects.

The patient underwent evaluation by the hematology-oncology team, who recommended a bone marrow biopsy. The biopsy revealed hypercellular marrow (100%) with prominent eosinophilic hyperplasia and maturing trilineage hematopoiesis without any evidence of myelofibrosis. It confirmed a diagnosis of hypereosinophilic syndrome with the FIP1L1‐PDGFRA fusion gene, indicating myeloid neoplasm with eosinophilia and PDGFR-A rearrangement. Subsequently, the patient underwent a cardiac MRI, which did not reveal any endomyocardial fibrosis or evidence of eosinophilic myocarditis. Treatment commenced with hydroxyurea, tapering of high-dose methylprednisolone, and initiation of a course of imatinib at 400 mg. Apixaban was prescribed for the DVT. Remarkably, within 16 days of admission, his absolute eosinophil count plummeted to 0.04 x 10^9^/L, indicating a robust response to treatment. However, a notable complication during his hospitalization was the onset of agitation, raising concerns of delirium. Following consultation with psychiatry, he was initiated on quetiapine and olanzapine, later transitioning to haloperidol.

A follow-up MRI of the brain conducted 22 days post-admission revealed evolving foci of diffusion restriction in the bilateral centrum semiovale and the splenium of the corpus callosum. Interestingly, the lesions in the infratentorial area were no longer evident, suggesting a favorable response to treatment. Concurrently, his neurological symptoms demonstrated significant improvement. Upon discharge, he was prescribed a daily dose of 100 mg of imatinib.

Subsequent follow-ups indicated the patient's continued tolerance to imatinib, with no recurrence of elevated eosinophil levels or emergence of new neurological symptoms.

## Discussion

HES exhibits a higher prevalence among males, with estimates suggesting a ratio of 9:1 [6]. Although rare, its reported incidence stands at 0.036 per 100,000 individuals, though population-level data are lacking [7]. Typically, patients present during adulthood, spanning ages 20-50, although pediatric cases have also been documented [8]. A study described the criteria for diagnosing HES, which include eosinophilia exceeding 1.5 × 10^9^/L persisting for more than six months, exclusion of other known causes of eosinophilia, and the presence of organ dysfunction directly attributable to eosinophils [2].

HES is classified into idiopathic, reactive type (induced by cytokines, resulting in polyclonal eosinophilia) and myeloproliferative type (characterized by genetic rearrangements, leading to monoclonal hypereosinophilia) [9]. Among these genetic anomalies is the deletion in chromosome 4q12, resulting in the FIP1L1-PDGFRA fusion protein, as seen in our patient. This mutation prompts a constitutively active tyrosine kinase, rendering imatinib a viable treatment option [10]. While the precise incidence of this mutation remains elusive, studies indicate its prevalence in a significant subset of patients with idiopathic hypereosinophilia [10]. Our patient exhibited a remarkable response to imatinib therapy, following an initial regimen of high-dose steroids and hydroxyurea, consistent with standard treatment protocols. Notably, the patient's mental status significantly improved post initiation of imatinib, alleviating a state of delirium. Similar cases have reported psychotic symptom amelioration with imatinib, suggesting that the patient's delirium stemmed directly from HES rather than as a consequence of stroke or hospitalization [11].

While diagnosis typically requires an eosinophil count exceeding 1.5 x 10^9^/L on two consecutive measurements, HES presents with a wide array of symptoms. Although multiple organ systems can be affected, cardiac manifestations are frequently observed, often resulting in restrictive cardiomyopathy or ventricular failure due to fibrosis [12]. Remarkably, our patient presented an atypical case as he lacked cardiac symptoms, and both transthoracic and transesophageal echocardiograms revealed no abnormalities. In certain instances, strokes in HES are categorized as cardioembolic, particularly those affecting watershed areas [13]. This is typically attributed to eosinophilic myocarditis. However, in our patient's case, the absence of cardiac abnormalities on echocardiograms suggests an alternative mechanism. HES involves manifestations in various organs, including the skin (causing angioedema, erythematous, and/or urticarial lesions, purpura, pruritus, Raynaud's phenomenon, and nail-fold splint hemorrhages), the gastrointestinal tract (presenting symptoms such as abdominal pain, diarrhea, ascites, colitis, enteritis, pancreatitis, and cholangitis), the pulmonary system (exhibiting symptoms such as cough, bronchial hyperreactivity, and pulmonary infiltrates), and hematological abnormalities (such as hepatomegaly, splenomegaly, anemia, thrombocytopenia, and arterial or venous thrombosis) [2]. Despite the identification of a femoro-popliteal DVT, the lack of a patent foramen ovale excludes it as the source of emboli. It is more plausible that his strokes resulted from blood hyperviscosity or hypercoagulability [14].

Even though neurologic manifestations of HES appear infrequent, an extensive literature review estimates their occurrence in only about 4% of HES patients [15]. Among these, ischemia and paresthesias of the upper limbs were the most commonly reported symptoms. In a literature review conducted by Ono et al. [14], it was found that 80% of cases (78 out of 97 cases) of strokes associated with HES were characterized by infarcts. Our patient, like others, exhibited an excellent response to therapy, with symptoms entering remission upon improvement in eosinophil counts [15]. The FIP1L1-PDGFRA fusion gene, identified in our patient, has been particularly linked to strokes of non-embolic origin, with a low recurrence rate observed after initiation of imatinib [16]. Interestingly, there is no discernible pattern in the brain territory affected, with case reports demonstrating isolated involvement of the middle cerebral artery [17], watershed infarcts [16], or diffuse bilateral involvement akin to our case [18]. In cases of HES, cerebral infarcts are commonly attributed to cardioembolism, with two main hypotheses proposed. The first involves the formation of intracardiac thrombi secondary to endomyocardial fibrosis, diagnosed using cardiac MRI. The second hypothesis, known as the "washout" theory, suggests reduced emboli clearance in hypoperfused vascular territories. Factors such as stenotic lesions, high blood cell counts (e.g., in polycythemia vera), and elevated eosinophil counts contribute to increased blood viscosity and hypercoagulability, potentially leading to atypical stroke patterns in compromised vascular territories [19].

## Conclusions

To our knowledge, there are no data comparing the incidence of thromboembolic and non-thromboembolic strokes in HES, although both mechanisms are conceivable. With this report, we aim to underscore the association between HES and strokes of unexplained origin, emphasizing the importance of revisiting basic laboratory tests if the standard stroke work-up fails to reveal a source. In such cases, particularly when hypereosinophilia is evident, an urgent bone marrow biopsy and hematologic evaluation are warranted, given the favorable response of HES patients to chemotherapy.
